# Survival analysis of pulpectomy in primary molars performed under dental general anaesthesia: a two-year retrospective study

**DOI:** 10.1186/s12903-022-02553-z

**Published:** 2022-12-10

**Authors:** Yongting Xie, Yan Wang, Qizhao Ma, Jing Li, Yandi Chen, Ran Yang, Ruijie Huang, Qiong Zhang, Jing Zou

**Affiliations:** State Key Laboratory of Oral Diseases & National Clinical Research Center for Oral Diseases, Department of Paediatric Dentistry, West China Hospital of Stomatology, Sichuan University, Chengdu, China

**Keywords:** Primary molars, Pulpectomy, Mineral trioxide aggregate, Survival analysis, Success rate

## Abstract

**Background:**

To retrospectively investigate the success rate of primary-molar pulpectomy performed under general anaesthesia and the potential risk factors that affect the 24-month success rate.

**Methods:**

The case data and two-year follow-up records of children (aged 3–6 years) who received pulpectomy in primary molars performed under general anaesthesia were reviewed and assessed. Potential risk factors included age, gender, decayed-missing-filled teeth, endodontic diagnosis, tooth location, and postobturation sealing of the pulp chamber floor with MTA. With a two-year follow-up period, the outcomes of all the primary molars were classified into success and failure. Survival analysis was used to assess the outcomes. The Kaplan–Meier method was used to analyse the success rate. Univariate and multivariate Cox proportional hazards regression models were used to evaluate the potential risk factors associated with the overall survival of primary molars.

**Results:**

A total of 410 teeth from 163 children (88 boys and 75 girls) were included in this study. The overall two-year success rate was 66.1% for all primary molars. The mean overall survival time for this study was 22.1 (95% CI, 21.73‒22.48) months. Multivariate Cox regression analysis demonstrated that endodontic diagnosis (irreversible pulpitis or periapical periodontitis), tooth location (maxillary or mandibular primary molar), and postobturation sealing of the pulp chamber floor (MTA or no-MTA) were significant risk factors for overall survival in this study (*P* < .05). The differences in success rates were not statistically significant in terms of age, gender, and decayed-missing-filled teeth (*P* > .05).

**Conclusions:**

When compared to teeth diagnosed with irreversible pulpitis, those with periapical periodontitis failed more frequently. Postobturation sealing of the pulp chamber floor with MTA improved the success rate of pulpectomy in primary molars, especially when the inflammation did not spread to the periradicular area.

## Background

According to the 4th National Oral Health Survey report in mainland China in 2017, the caries prevalence rates in 3- and 5-year-old children were as high as 51% and 71%, respectively [1]. Inflammation of dental pulp tissue, known as pulpitis, which results from harmful stimuli, such as bacterial infection of the tooth structure. Carious pulp exposure and preoperative spontaneous evoked pain are seen as signs of irreversible pulpitis [2]. Periapical periodontitis is an inflammatory lesion around the apex of a tooth root and can be considered a sequel to tooth decay, irreversible pulpitis, and pulp necrosis. Pulpitis and periapical periodontitis caused by severe dental caries are the most common reasons for the early loss of primary teeth. Compared to tooth extraction, pulpectomy is considered to be more conservative. Therefore, improving the success rate of pulpectomy to maintain the integrity of dentition has become a hot issue for paediatric dentists.

Pulpectomy is indicated in a primary tooth with symptoms and signs indicative of irreversible pulpitis or necrosis [3, 4]. The failure of pulpectomy in primary teeth results mainly from incomplete removal of infected tissue and the presence of a complicated root canal system, especially in primary molars [5–7]. Due to its close anatomical relationship with the dental follicle of the permanent successor tooth, the furcation region of primary molars is important [8]. Because of the special anatomical structure of primary molars, a radiolucent lesion in primary molars is unlikely to be found in the periapical region; instead, it is found in the interradicular region. Previous investigators employed different root canal irrigation fluids [9], root filling materials [10, 11], root canal preparation instruments [12], and root length determination methods [13] to improve the success rate of pulpectomy in primary teeth. However, a recent systematic review revealed that there is no conclusive evidence that one medicament or approach was better than another; hence, the choice of medicament remained at the clinician’s discretion when performing pulpectomy in primary teeth [14]. Therefore, it is important to study the potential risk factors that affect the success rate of pulpectomy in primary molars and find a new technology to improve the success rate.

Mineral trioxide aggregate (MTA) is a dental material with a gel pH of 12.5, mainly consisting of lime, silica, and bismuth oxides. When exposed to moisture and allowed to be set for approximately 4 h, MTA hardens into a stiff mass [15, 16]. It has superior biocompatibility, bactericidal effects, and the ability to induce cementogenesis [17]. A systematic review showed that MTA seemed to be the best material (in terms of biocompatibility and effectiveness) to place over the pulp tissues after pulpotomy [14]. Another study found that MTA pulpotomies for carious exposed teeth had a 100% success rate at the end of the 24-month follow-up period [18]. MTA has been proven to stimulate hard tissue healing when used to treat internal resorption and furcal perforations and to be more effective, due to its sealing ability, when used in root canal treatment of primary second molars without successors [19]. Because of these advantages, we speculated that postobturation sealing of the pulp chamber floor with MTA would improve the success rate of pulpectomy in primary molars. This retrospective study aimed to investigate the success rate of pulpectomy for primary molars performed under general anaesthesia (DGA) and the potential risk factors that affect the 24-month success rate.

## Methods

### Sample selection

Data were obtained from electronic medical records for the period between February 2015 and May 2019, which covered the details of oral examinations, diagnoses and radiographic examinations. Children (aged 3–6 years) undergoing dental treatment under DGA in the Department of Paediatric Dentistry, West China Hospital of Stomatology, Sichuan University, were included according to the inclusion criteria in this study. The inclusion criteria included the following: (1) Medically healthy children with complete medical records at treatment and during follow-up visits. (2) Children without congenital, developmental, or acquired dental anomalies (e.g., microdontia, short roots, or congenitally absent succedaneous teeth). (3) Primary molars diagnosed with irreversible pulpitis, pulp necrosis, or periapical periodontitis. (4) Pre-treatment radiograph showing no radiographic evidence of pulp calcification or internal resorption, roots exhibiting minimal or no resorption and intact succedaneous tooth germ. (5) Pulpectomy was performed using the same filling materials and a stainless-steel crown as a final restoration for those that received pulpectomy.

### Data collection

**Fig. 1:**
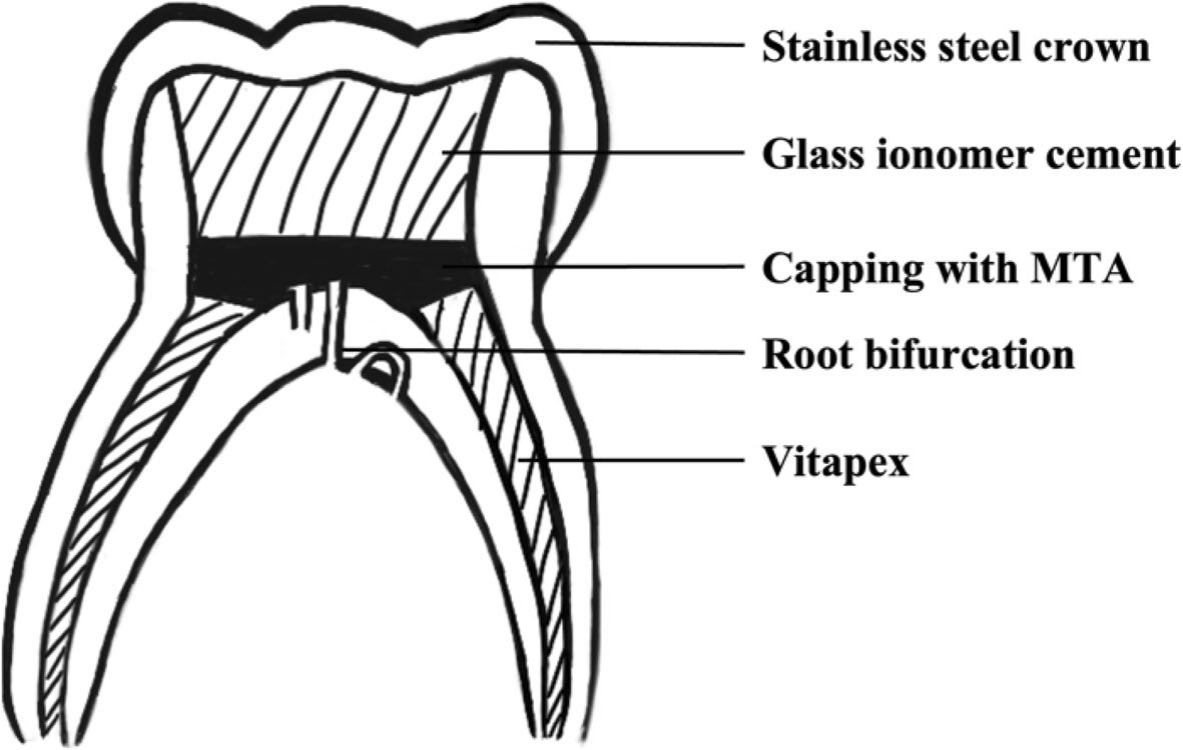
Schematic diagram of a primary molar in the MTA group

Data collected included the patient’s dental records, DGA-related documents and medical history in both electronic and paper forms. Potential risk factors included gender, age at the time of treatment (in months), decayed-missing-filled teeth (dmft), endodontic diagnosis (irreversible pulpitis or periapical periodontitis), tooth location (maxillary or mandibular primary molar), and postobturation sealing of the pulp chamber floor (MTA or no-MTA). The study relied on the diagnosis listed in the patient’s files and the teeth diagnosed with pulp necrosis were added to the irreversible pulpitis group. In the MTA group, after the root canals were filled with Vitapex (Neo Dental Chemical Products Co, Ltd, Tokyo, Japan), postobturation sealing of the pulp chamber floor was accomplished with a 2-mm layer of MTA (Dentsply Tulsa Dental Specialties, Inc, Johnson city, TN), and then the teeth were then restored with GC Fuji restorative cement gold label type 9 (GC Fuji Co, Ltd, Tokyo, Japan) (Fig. 1). In the no-MTA group, after root canal obturation with Vitapex, the pulp chamber was restored with cement gold label type 9. In both groups, all molars were restored with stainless steel crowns during the same visit.

Clinical examinations were performed every three months, and radiographic examinations were performed every six months of the follow-up period. The clinical examinations included inspection, exploration, palpation, percussion, and tooth mobility assessment. The outcomes of pulpectomy in primary molars were determined based on clinical and radiographic examinations. The criteria for success were, teeth without symptoms and signs, including pain, abscess, fistula, and pathological loosening of teeth in clinical examinations and the absence of pathological internal or external root absorption and presence/aggravation of periapical or furcal radiolucency in radiographic examinations.

Evaluations were performed by three certified paediatric dentists who trained and calibrated clinically and radiographically to carry out the follow-up. When evaluation results differed between the examiners, the majority opinion was accepted. The interexaminer reliability for clinical and radiographic examinations was calculated by Cohen’s kappa statistic (1 and 0.78, respectively).

### Statistical analysis

The overall survival (OS) time was calculated from the date of treatment (in months) to the date of failure. Mean survival time (MST) was expressed as the mean OS time (± standard deviation). Successfully treated teeth that lasted until the closing date of the study were considered censored data. The data were analysed using SPSS 26.0. The survival rate was compared between the two groups using the chi-squared test. A Kaplan–Meier plot was used to estimate the overall survival curves. The survival curves of the two groups were compared using the log-rank test. Univariate and multivariate Cox proportional hazards regression models were used to evaluate potential risk factors for survival. The hazard ratio (HR) is the ratio of the hazard rates corresponding to the risk of failure. In all instances, *P* < 0.05 was considered statistically significant.

## Results

### Patients and tooth characteristics

A total of 410 teeth from 163 children (88 boys and 75 girls) were included in the present study. The mean age of patients at the time of treatment was 55.80 ± 9.68 months, and the mean dmft was 16.50 ± 3.27. After the two-year follow-up period, 271 (66.1%) pulpectomies were considered successful, and 139 (33.9%) were considered failure (Table 1).

**Table 1:** Descriptive statistics of the patient population and subgroups

Variable	All teeth *n* = 410	Success *n* = 271	Failure *n* = 139
**Age, mean (SD)/mo**	55.80 (9.68)	55.32 (9.43)	56.75 (10.07)
**Gender, n (%)**			
Male	230 (56.1)	151 (65.65)	79 (34.35)
Female	180 (43.9)	120 (66.64)	60 (33.33)
**dmft, mean (SD)**	16.50 (3.27)	16.48 (3.27)	16.54 (3.27)
**Endodontic diagnosis, n (%)**			
Irreversible pulpitis	269 (65.61)	210 (78.07)	59 (21.93)
Periapical periodontitis	141 (34.39)	61 (43.26)	80 (56.73)
**Tooth location, n (%)**			
Maxillary primary molar	142 (34.63)	105 (73.94)	37 (26.06)
Mandibular primary molar	268 (65.37)	166 (61.94)	102 (38.06)
**Postobturation sealing materidal, n (%)**			
MTA	238 (58.05)	183 (76.89)	55 (23.11)
No-MTA	172 (41.95)	88 (51.16)	84 (48.84)

*SD* standard deviation, *mo* month, *dmft* decayed‐missing‐filled‐teeth, *MTA* mineral trioxide aggregate

### Survival and recurrence

**Fig. 2:**
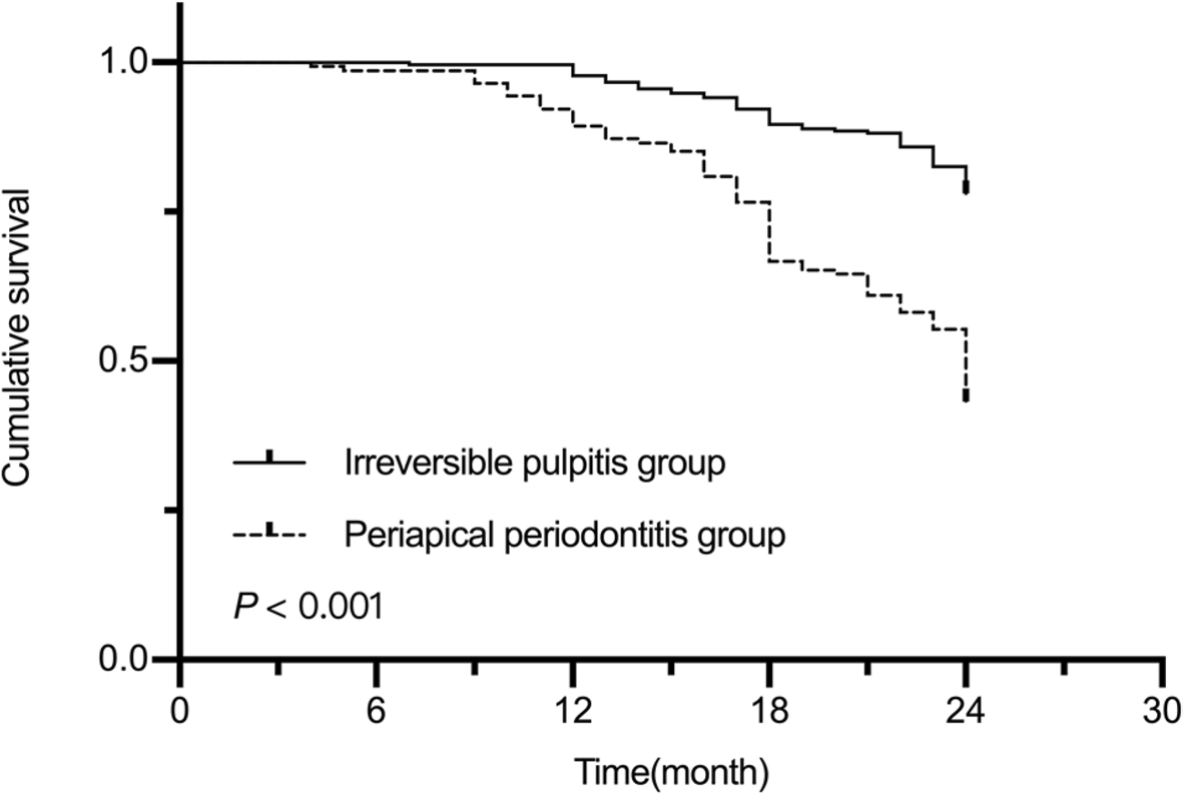
Survival curves for the irreversible pulpitis group and the periapical periodontitis group

**Fig. 3:**
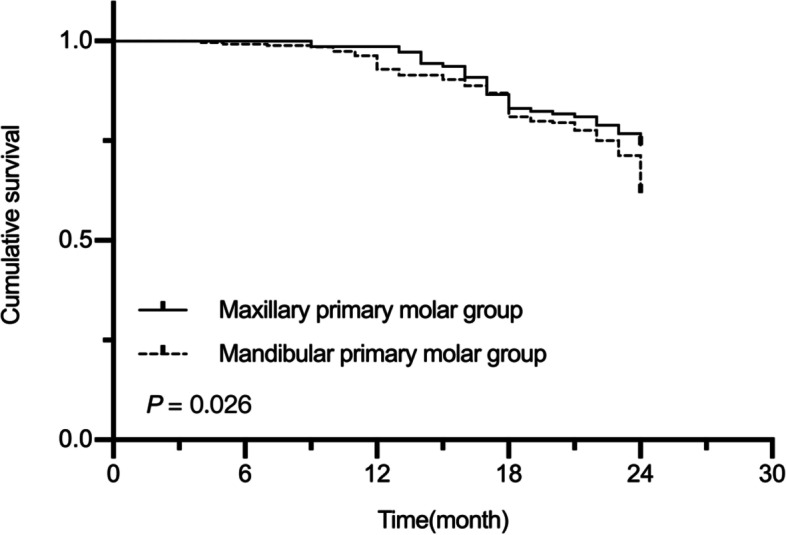
Survival curves for the maxillary primary molar group and the mandibular primary molar group

**Fig. 4:**
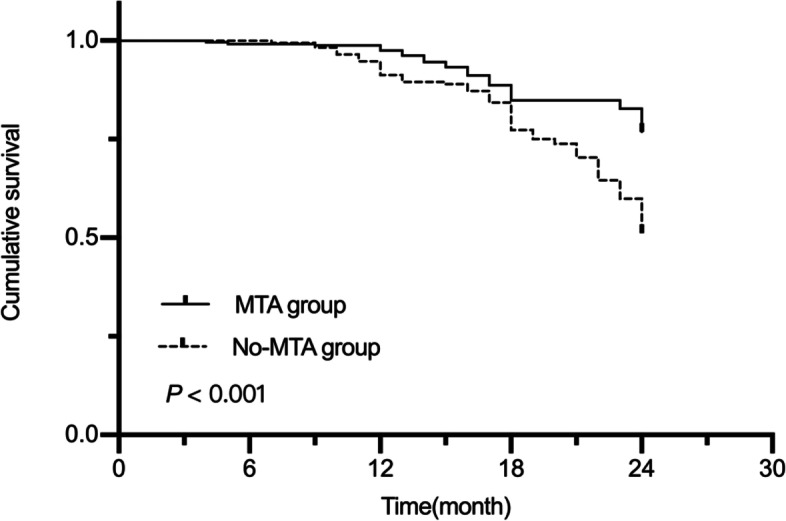
Survival curves for the MTA group and the no-MTA group

The two-year success rate was 78.07% for the irreversible pulpitis group and 43.26% for the periapical periodontitis group (*P* < 0.001). For maxillary molars, the two-year success rate was 73.94%, while for the mandibular molars it was 61.94%, and this difference was statistically significant (*P* = 0.009). The two-year success rate was 76.89% for the MTA group and 51% for the no-MTA group (*P* < 0.001). The MST for this study was 22.10 (95% CI, 21.73‒22.48) months. The MST was 22.93 (95% CI, 22.58‒23.28) in the irreversible pulpitis group and 20.53 (95% CI, 19.72‒21.34) in the periapical periodontitis group. The MST was 22.41 (95% CI, 21.85‒22.97) in the maxillary primary molars and 21.94 (95% CI, 21.45‒22.43) in the mandibular primary molars. The MST was 22.61 (95% CI, 22.16‒23.08) in the MTA group and 21.4 (95% CI, 20.77‒22.04) in the no-MTA group. Kaplan–Meier analysis showed a longer OS in the irreversible pulpitis group than in the periapical periodontitis group (*P* < 0.001, Fig. 2). The maxillary primary molars also had a longer OS than the mandibular primary molars (*P* = 0.026, Fig. 3). Moreover, the MTA group had a longer OS than the no-MTA group (*P* < 0.001, Fig. 4).

### Cox regression analysis of failure

Potential risk factors including age at the time of treatment (in months), gender, dmft, endodontic diagnosis (irreversible pulpitis or periapical periodontitis), tooth location (maxillary or mandibular primary molar), and postobturation sealing of the pulp chamber floor (MTA or no-MTA) were analysed by univariate and multivariate Cox proportional hazards regression models. Multivariate Cox proportional hazards regression analysis showed that endodontic diagnosis (irreversible pulpitis or periapical periodontitis), tooth location (maxillary or mandibular primary molar), and postobturation sealing of the pulp chamber floor with MTA were significant risk factors for OS in this study population (Table 2). The differences in success rate in terms of age, gender, and dmft were not statistically significant (*P* > 0.05). Compared with irreversible pulpitis, periapical periodontitis exhibited a higher risk of failure; the HR (95% CI) was 3.28 (2.34‒4.59) in a univariate model *(P* < 0.001) and 2.76 (1.92‒3.99) after adjusting for other risk factors (*P* < 0.001). Moreover, compared with maxillary primary molars, mandibular primary molars had a higher risk of failure; the HR (95% CI) was 1.52 (1.04‒2.21) in a univariate model *(P* = 0.030) and 1.47 (1.00‒2.16) after adjusting for other risk factors (*P* = 0.047). The MTA group was associated with a 25.73% decrease in the risk of failure (HR, 2.44; 95% CI, 1.73‒3.42; *P* < 0.001). The benefit of postobturation sealing of the pulp chamber floor with MTA remained significant after adjusting for other risk factors in multivariate analysis (HR, 1.85; 95% CI, 1.29‒2.63; *P* = 0.001).

**Table 2:** Univariate and multivariate Cox regression models for risk factors that could have influenced the survival rate of primary molar pulpectomy

Variable	Univariate		Multivariate	
	HR (95%CI)	*P*	HR (95%CI)	*P*
**Age**	1.01 (1.00–1.03)	0.159	1.00 (0.98–1.02)	0.719
**dmft**	1.00 (0.95–1.05)	0.935	0.98 (0.93–1.03)	0.442
**Gender**		0.749		0.614
Male	1(Indicator)		1(Indicator)	
Female	0.95 (0.68–1.32)		1.09 (0.77–1.54)	
**Endodontic diagnosis**		** < 0.001**		** < 0.001**
Irreversible pulpitis	1(Indicator)		1(Indicator)	
Periapical periodontitis	3.28 (2.34–4.60)		2.76 (1.92–3.99)	
**Tooth location**		**0.030**		**0.047**
Maxillary primary molar	1(Indicator)		1(Indicator)	
Mandibular primary molar	1.52 (1.04–2.21)		1.47 (1.00–2.16)	
**Postobturation sealing materidal**		** < 0.001**		**0.001**
MTA	1(Indicator)		1(Indicator)	
No-MTA	2.44 (1.73–3.42)		1.85 (1.29–2.63)	

*HR* hazard ratio, *CI* confidence interval, *dmft* decayed‐missing filled‐teeth, *MTA* mineral trioxide aggregate

To verify the superiority of postobturation sealing of the pulp chamber floor with MTA in pulpectomy procedures, we further analysed the use and outcome of MTA in the irreversible pulpitis and periapical periodontitis groups (Table 3). In the irreversible pulpitis group, MTA was used to postobturation sealing of the pulp chamber floor in 180 teeth, while MTA was not used in 89 teeth. After two years of follow-up in the MTA group, 154 (85.56%) teeth were successful, and 26 (14.44%) teeth failed. In the no-MTA group, 56 (62.92%) teeth were successful, and 33 (37.08%) failed. For teeth diagnosed with irreversible pulpitis, differences in the success rates between the two groups were statistically significant (*P* < 0.001).

**Table 3:** The success/failure rates of the two postobturation sealing materials in primary molars diagnosed with irreversible pulpitis or periapical periodontitis

Variable	All teeth *n* = 410	Success *n* = 271	Failure *n* = 139	*P*
**Irreversible pulpitis, n (%)**				** < 0.001**
MTA	180	154 (85.56)	26 (14.44)	
No-MTA	89	56 (62.92)	33 (37.08)	
**Periapical periodontitis, n (%)**				0.177
MTA	58	29 (50)	29 (50)	
No-MTA	83	32 (38.55)	51 (61.45)	

*MTA* mineral trioxide aggregate

MTA was used for postobturation sealing of the pulp chamber floor in 58 teeth, while MTA was not used in 83 teeth. After two years of follow-up, 29 (50%) teeth in the MTA group were considered successful, and 29 (50%) teeth were considered failure. In the no-MTA group, 32 (38.55%) teeth were considered successful, and 51 (61.45%) were considered failure. The differences in the success rates in the periapical periodontitis groups were not statistically significantly different (*P* > 0.05).

## Discussion

Improving the success rate of pulpectomy is always a hot issue for paediatric dentists. Many factors affect the success of pulpectomy in primary molars due to the complexity of the root canal system and individual differences between patients. This study demonstrated that endodontic diagnosis (irreversible pulpitis or periapical periodontitis), tooth location (maxillary or mandibular primary molar) and postobturation sealing of the pulp chamber floor with MTA were significant risk factors for OS in this study (*P* < 0.05). Age and dmft were not risk factors that affected the 24-month success rate in this study. This may be related to all children who underwent pulpectomy under DGA having a similar age and dmft.

Most previous reports suggested that the success rate and prognosis of pulpectomy for teeth with irreversible pulpitis were better than those with periapical periodontitis, which was in accordance with this present study [9, 20, 21]. In this study, the two-year success rate of pulpectomy for teeth with irreversible pulpitis was 78.07% compared to 43.26% for primary molars diagnosed with periapical periodontitis. An earlier study [20] found that the primary molars’ pulpectomy success rate was 74.71% for teeth diagnosed with pulpitis and 63.64% for teeth diagnosed with periapical periodontitis. However, that study only included 22 primary molars with periapical periodontitis the sample size of which was less than ours. We speculate that compared to the teeth diagnosed with irreversible pulpitis, the pathogenic factors in the root canal system of teeth with periapical periodontitis were more invasive, which could be the main cause of failure. It has been previously reported that symptomatic teeth with necrotic pulps and periapical bone loss frequently harbour many microbiotas in root canals, many of which have more complicated anaerobic bacterial flora [22]. Bacteria and their metabolites can stimulate the cell- and humoral-mediated immune responses in periapical lesions to trigger the production of the cytokines interleukin-1 (IL-1) and tumour necrosis factor (TNF), which cause bone destruction [23]. The periodontium is anatomically interrelated with the dental pulp through lateral, apical, and furcal foramina, providing a channel for the interchange of noxious agents between the two tissue compartments when one or both are damaged. In addition, the vertical distance between the marginal gingiva and root bifurcation area of primary molars is short, making it easier to aggravate the lesion through the gingival sulcus. This may be the reason for the poor prognosis of pulpectomies performed on teeth diagnosed with periapical periodontitis.

Some studies have compared the prognosis of pulpectomy in mandibular and maxillary primary molars, and authors have reported a higher failure rate of pulpectomy in mandibular primary molars than in maxillary primary molars [24, 25]. Similarly, our results showed a significantly higher success rate of pulpectomy in maxillary primary molars than in mandibular primary molars. However, other studies found no significant differences between them [26, 27]. The differences in success rates of pulpectomy between mandibular and maxillary primary molars have not been clearly established, which may be attributed to the differences in the complexity of the root canal system. The anatomy of the root canal system of primary molars and the number and type of accessory root canals vary significantly. In addition, it is usually difficult to observe the initial radiographic changes in bone density between or around the roots of the maxillary molars while the permanent tooth germ and roots overlap the interradicular bone. However, this is not a good reason for undertaking three-dimensional radiographic examinations.

This study proposed postobturation sealing of the pulp chamber floor to prevent communication between the root bifurcation and the pulp chamber and observed whether this would improve the success rate of pulpectomy in primary molars. In the current study, teeth in the MTA group had a significantly lower risk of failure than those in the no-MTA group in a multivariate model. To verify the superiority of postobturation sealing of the pulp chamber floor with MTA in pulpectomy, we further analysed the effect of MTA in the irreversible pulpitis and periapical periodontitis groups. The difference in success rate was statistically significant in the irreversible pulpitis group, while it was not significantly different in the periapical periodontitis groups. This may be related to the damage to periodontal tissue caused by periapical periodontitis, which is difficult to repair. The success rate and prognosis of pulpectomy for periapical periodontitis were worse than those of irreversible pulpitis. More clinical samples are necessary for further analyses.

MTA has several desirable properties in terms of biocompatibility, bioactivity, hydrophilicity, radiopacity, sealing ability and low solubility [28–30]. In dentistry, two of the most important properties are its sealing ability and biocompatibility. The seal is achieved through its similar expansion and contraction properties to dentin, which results in high resistance to marginal leakage [31]. Postobturation sealing of the pulp chamber floor with MTA sealed the communication between the root bifurcation tissues and the pulp chamber and blocked the spread of inflammation to the periradicular area. On the other hand, the high biocompatibility of MTA promotes optimum healing responses. This has been shown histologically in the periradicular tissue area with the formation of new cementum [32] and in the pulp space with a modest inflammatory response and bridge creation [33]. In addition, when MTA is in contact with moisture, its main component, calcium oxide, converts into calcium hydroxide. Due to this conversion, a microenvironment with a high pH promotes desirable antibacterial properties [34]. However, the discolouration potential of teeth treated with MTA and the difficulty of root canal retreatment are considered some of the disadvantages of this material. In this study, the success rate and prognosis of pulpectomy for teeth with periapical periodontitis were worse than those diagnosed with irreversible pulpitis. This may be related to the damage to periodontal tissue caused by periapical periodontitis, which is difficult to repair. More studies are needed to explore this theory.

Postobturation sealing of the pulp chamber floor with MTA and blocking the communication between the root bifurcation and pulp chamber to improve the success rate of pulpectomy in primary molars have not been reported before. This study provides a new potential orientation to improve the success rate of pulpectomy in primary molars. However, this was a retrospective study. The diagnosis of pulp status was made by different treating dentists. In addition, the quality of the root canal filling was not included in this study and the radiographic examination was not standardized.

## Conclusions

When compared to teeth diagnosed with irreversible pulpitis, those with periapical periodontitis failed more frequently. Postobturation sealing of the pulp chamber floor with MTA improved the success rate of pulpectomy in primary molars, especially when the inflammation did not spread to the periradicular area. The findings suggest this method could be used to improve the success rate of pulpectomy in primary molars.

## Data Availability

All data generated and analysed in this study are included within the article or available from the corresponding author upon reasonable request.

## Abbreviations

MTA: Mineral trioxide aggregate
OS: Overall survival
MST: Mean survival time
DGA: Dental general anaesthesia
dmft: Decayed-missing-filled teeth
HRs: Hazard ratios
95% CI: 95% Confidence intervals
SD: Standard deviation
mo: Month
IL-1: Interleukin-1
TNF: Tumour necrosis factor
